# Culture Conversion Rate at 2 Months of Treatment According to Diagnostic Methods among Patients with Culture-Positive Pulmonary Tuberculosis

**DOI:** 10.1371/journal.pone.0103768

**Published:** 2014-08-08

**Authors:** Ha Youn Lee, Kyoung Ok Chae, Chang Hoon Lee, Sun Mi Choi, Jinwoo Lee, Young Sik Park, Sang-Min Lee, Chul-Gyu Yoo, Young Whan Kim, Sung Koo Han, Jae-Joon Yim

**Affiliations:** Division of Pulmonary and Critical Care Medicine, Department of Internal Medicine, Seoul National University College of Medicine, Seoul, Republic of Korea; Glaxo Smith Kline, Denmark

## Abstract

**Introduction:**

The culture-negative conversion rate of sputum after 2 months of treatment in patients with pulmonary tuberculosis (TB) is used as a reliable surrogate marker for relapse after completion of treatment. We hypothesized that culture conversion of sputum at 2 months of anti-TB treatment and the time to culture conversion are different among pulmonary TB patients who are diagnosed using different methods.

**Methods:**

Culture-confirmed pulmonary TB patients who were diagnosed between 1 January, 2011 and 31 December, 2012 were classified into three groups based on the diagnostic method that prompted treatment initiation: positive acid-fast bacilli (AFB) staining of sputum (smear-positive group), negative AFB staining, but *Mycobacterium tuberculosis* was cultured from sputum (culture-positive group), and positive AFB staining, positive polymerase chain reaction (PCR) for *M. tuberculosis*, or culture of *M. tuberculosis* from a bronchoscopic specimen (bronchoscopy group). Rates of negative mycobacterial culture conversion at 2 months of anti-TB treatment and the time to negative culture conversion of sputum were compared among the three groups.

**Results:**

A total of 203 patients with culture-confirmed pulmonary TB were included in the final analysis. TB patients in the culture-positive group (94.1%) and the bronchoscopy group (97.6%) showed a higher culture conversion rate at 2 months of treatment than those in the smear-positive group (78.7%, P = 0.001). Additionally, the time to culture conversion was longer in the smear-positive group (median, 40 days) than in the culture-positive (median, 19 days; P = 0.009) and bronchoscopy groups (median, 29 days; P = 0.004).

**Conclusions:**

The higher culture conversion rate at 2 months and the shorter time to culture conversion among pulmonary TB patients with a negative AFB smear suggests the feasibility of shortening treatment duration and isolation in these patients.

## Introduction

Tuberculosis (TB) is a great challenge to the maintenance of human health. According to the World Health Organization (WHO), an estimated 8.6 million new cases of TB developed and 1.3 million people died from TB worldwide in 2012 [1]. Over the past several decades, constant efforts to achieve a successful treatment of TB have been attempted. Based on several clinical trials, the recommended treatment regimen for pulmonary TB caused by drug-susceptible organisms is combination therapy consisting of 2 months of intensive phase and 4 months of continuation phase therapy [2]. Intensive phase treatment consists of isoniazid, rifampicin, pyrazinamide, and ethambutol. This treatment has been reported to be effective, and is known to have a 70–95% sputum-negative conversion rate after 2 months, 62.3–88% cure rate, and 0–3.4% relapse rate [3]–[7].

According to a recent WHO report, globally, the treatment success rates among the 2.6 million patients with new cases of pulmonary TB who were treated in 2011 was 87% [1]. To monitor outcomes of treatment, sputum is recommended to be obtained for microscopic examination and culture at monthly intervals until two consecutive culture specimens are negative. In particular, the sputum culture-negative conversion rate after 2 months of anti-TB treatment is a reliable surrogate marker for relapse after completion of treatment [8]. Cavitary lesions, a high pre-treatment smear grade, smoking, uncontrolled diabetes, a past history of TB, and time to detection of *Mycobacterium tuberculosis* in initial sputum culture are factors that are independently associated with a delay in culture conversion [9]–[13]. However, the effect of diagnostic methods on the rate of negative culture conversion at 2 months of treatment has not been determined yet.

This study aimed to test the hypothesis that pulmonary TB patients who have negative findings on a smear or are diagnosed using bronchoscopic specimens have a higher culture conversion rate at 2 months of treatment and a shorter time to negative conversion than those who are positive for acid-fast staining of sputum.

## Materials and Methods

### Ethics statement

The protocol of this study was approved by the ethical review committee of Seoul National University Hospital. No consent was obtained because patients' records and information were anonymized and de-identified prior to analysis.

### Study design and patients

A retrospective cohort study was performed, including pulmonary TB patients diagnosed based on culture of *M. tuberculosis* using sputum or bronchoscopic specimens. Culture-confirmed pulmonary TB patients who were diagnosed between 1 January, 2011 and 31 December, 2012 at Seoul National University Hospital were included for analysis.

TB patients were excluded from the analysis if resistance to any anti-TB drug was identified, an anti-TB drug, except for isoniazid, rifampicin, pyrazinamide, and ethambutol, was used, or any anti-TB drug was stopped because of adverse drug events. Additionally, patients in whom culture conversion was not achieved until 8 or 9 weeks, but mycobacterial culture of sputum was not requested between 8 and 9 weeks of treatment, were excluded.

Information on smoking, co-morbidities, HIV status, diabetes, previous treatment for TB, radiographic findings, anti-TB treatment regimen, adverse drug events, serial results of acid-fast bacilli (AFB) staining, mycobacterial culture of sputum, and bronchoscopic specimens was collected.

### Classification of patients

Patients were classified into three groups based on the diagnostic method that prompted treatment initiation: 1) positive AFB staining of sputum (smear-positive group), 2) negative AFB staining, but *M. tuberculosis* was cultured from sputum (culture-positive group), and 3) positive AFB staining, positive polymerase chain reaction (PCR) for *M. tuberculosis*, or culture of *M. tuberculosis* from a bronchoscopic specimen (bronchoscopy group).

### Definitions and comparisons

In our hospital, three sets of AFB smears and mycobacterial culture are performed for those who are suspected as having TB, although sometimes this varies. Once anti-TB treatment is initiated, patients in our hospital are monitored by performing an AFB smear and culture using sputum at 1 or 2 weeks of treatment initiation, and then monthly until 6 months.

Negative culture conversion was defined as two consecutive negative sputum cultures on liquid medium. The date of culture conversion was defined as the date of the initial negative culture. Negative sputum culture followed by contaminated cultures was also regarded as culture conversion. If patients could not expectorate sputum after one occurrence of negative sputum culture, culture conversion was also defined.

The rate of negative mycobacterial culture conversion at 2 months of anti-TB treatment and the time to negative culture conversion of sputum were compared among the three groups. The culture conversion rate at 2 months was defined as negative culture conversion at 8 weeks (or 9 weeks if results at 8 weeks were unavailable). The time to culture conversion was defined as the number of days from initiation of anti-TB treatment to the date of culture conversion.

### Diagnostic methods

All of the specimens for AFB smears and cultures were pre-treated by decontamination with 4% (w/v) NaOH and centrifugation at 3000 × *g* for 20 min. The AFB smears were examined after auramine-rhodamine staining. Culture was considered positive if *M. tuberculosis* was cultured in liquid broth media. For the cultures, sediment was incubated in MGIT tubes (Becton, Dickinson and Co., Sparks, MD, USA) for 6 weeks. Bronchoscopy was performed in suspected pulmonary TB patients who could not expectorate sputum or in those with negative AFB staining of sputum if needed. For PCR of bronchial washing or fluid from bronchoalveolar lavage, the Amplicor *Mycobacterium tuberculosis* test (Roche Molecular Systems, Branchburg, NJ, USA) or Xpert MTB/RIF assay (Cepheid, Inc., Sunnyvale, CA, USA) was used.

### Statistical analysis

Baseline demographic and clinical characteristics were summarized using descriptive statistics, such as the median and interquartile range. For comparisons between groups, *P* values were reported by using Pearson's χ^2^ test or one way ANOVA if appropriate.

The association between diagnostic methods and culture conversion at 2 months of treatment was investigated by logistic regression. Unadjusted and adjusted odds ratios (ORs) after adjusting for age and sex are shown together with 95% confidence intervals (CIs) and *P* values. Cox proportional hazard regression was performed to compare the time to culture conversion between the three groups. All statistical analyses were performed using Stata version 12 (Stata Corp., College Station, TX, USA).

## Results

### Baseline characteristics of patients

During the study period, 366 patients were diagnosed as having culture-positive pulmonary TB. We excluded eight patients who had anti-TB drugs stopped because of adverse drug events, 16 patients who had resistance to any anti-TB drug, 101 patients in whom culture of sputum was not performed between 8 and 9 weeks of treatment, although culture conversion was not achieved until 9 weeks, and 38 who were transferred out or lost to follow-up. Of the 203 patients included in the final analysis, 61 were classified into the smear-positive group, 101 into the culture-positive group, and 41 into the bronchoscopy group. *M. tuberculosis* was cultured from bronchoscopic specimens in 40 out of the 41 TB patients in the bronchoscopy group. PCR for TB was positive in 27 patients, AFB smears using bronchoscopic specimens were positive in two patients. In addition, *M. tuberculosis* was also cultured from sputum at a later date in 15 patients.

The median age of patients in the smear-positive group and the culture-positive group was 58 years and that of patients in the bronchoscopy group was 54 years. Thirty-eight (62.3%) patients in the smear-positive group, 59 (58.4%) in the culture positive group, and 19 (46.3%) in the bronchoscopy group were men. There were no differences in body mass index, smoking status, or comorbidities between the three groups. Although a proportion of patients who had bilateral lesions involving TB were similar, patients with cavities were more common in the smear-positive group (smear-positive group = 41.0% vs. culture-positive group = 16.8% vs. bronchoscopy group = 22.0%, P = 0.003) (Table 1).

**Table 1:** Demographic and clinical characteristics of 203 patients with culture-confirmed pulmonary tuberculosis.

Characteristics	Smear-positive group (n = 61)	Culture-positive group (n = 101)	Bronchoscopy group (n = 41)	*P* value
Age (y), median (IQR)	58 (43, 70)	58 (40, 70)	54 (37, 62)	0.96
Male (%)	38 (62.3)	59 (58.4)	19 (46.3)	0.27
Body mass index (kg/m^2^), median (IQR)	20.6 (17.7, 23.5)	21.5 (19.5, 23.9)	21.3 (19.9, 22.4)	0.65
Past history of TB treatment (%)	9 (14.8)	20 (19.8)	9 (21.9)	0.52
Smoking status				
Current smoker (%)	10 (16.4)	15 (14.9)	7 (17.1)	0.34
Smoking history (pack-y), median (IQR)	20 (30, 50)	20 (20, 43)	15 (5, 50)	0.55
Comorbidities				
Malignancy (%)	5 (8.2)	14 (13.9)	1 (2.4)	0.11
Diabetes (%)	15 (24.6)	23 (22.8)	9 (22.0)	0.95
Chronic kidney disease (%)	1 (1.6)	5 (5.0)	1 (2.4)	0.60
Chronic liver disease (%)	4 (6.6)	5 (5.0)	2 (4.9)	0.92
HIV/AIDS (%)	1 (1.6)	0 (0.0)	0 (0.0)	0.5
Radiographic findings				
Presence of cavity (%)	25 (41.0)	17 (16.8)	9 (22.0)	0.003
Bilateral lesions (%)	27 (44.3)	31 (30.7)	13 (31.7)	0.19

Abbreviations: IQR; Interquartile range

### Culture conversion rate at 2 months of anti-TB treatment

Out of 203 patients, 183 patients (90.1%) achieved negative culture conversion of sputum at 2 months of anti-TB treatment. The culture conversion rate at 2 months in the smear-positive group (78.7%) was significantly lower than that in the other two groups (94.1% in the culture-positive group; 97.6% in the bronchoscopy group, P = 0.001). The ORs for culture conversion at 2 months in the culture-positive group and the bronchoscopy group were 4.29 (95% CI, 1.53–12.0) and 10.8 (95% CI, 1.36–86.4), respectively, compared with the smear-positive group. These results were not changed after adjusting for sex and age of the patients, with an OR for the culture-positive group of 4.21 (95% CI, 1.50–11.9) and that for the bronchoscopy group of 9.77 (95% CI, 1.21–78.7) compared with the smear-positive group (Table 2).

**Table 2:** Culture conversion rate at 2 months of treatment in TB patients diagnosed by different methods.

Characteristics	Smear-positive group (n = 61)	Culture-positive group (n = 101)	Bronchoscopy group (n = 41)
Culture conversion rates at 2 months of treatment	48 patients (78.7%)	95 patients (94.1%)	40 patients (97.6%)
OR (95% CI)	1 (Reference)	4.29 (1.53, 12.0)	10.8 (1.36, 86.4)
Adjusted OR* (95% CI)	1 (Reference)	4.21 (1.50, 11.9)	9.77 (1.21, 78.7)

Abbreviations: OR; odds ratio, CI; confidence interval.

*Adjusted for age and sex.

### Time to culture conversion

**Figure 1:**
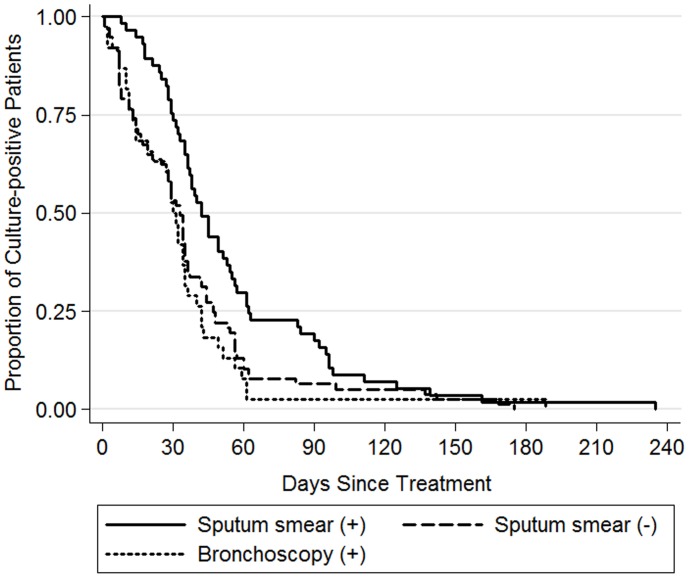
Comparison of time to culture conversion among TB patients diagnosed by different methods. P = 0.001 by log-rank test.

Among all of the 203 patients, the median time to culture conversion was 31 days (interquartile range: 7, 47). The time to culture conversion was significantly longer in the smear-positive group (median, 40 days) than in the culture positive group (median, 19 days; P = 0.009) and the bronchoscopy group (median, 29 days; P = 0.004). These results were unchanged after adjusting for sex and age of the patients (Table 3). The time to culture conversion was demonstrated in Kaplan–Meier survival analysis (Figure 1).

**Table 3:** Time to culture conversion in TB patients diagnosed by different methods.

Characteristics	Smear-positive group (n = 61)	Culture-positive group (n = 101)	Bronchoscopy group (n = 41)
Time to culture conversion, Median (IQR)	40 (28, 61)	19 (1,41)	29 (11, 40)
*P* value from comparison with smear-positive group, unadjusted	-	0.009	0.004
*P* value from comparison with positive AFB smear group, adjusted for age and sex	-	0.008	0.009

## Discussion

In this study, we showed that culture conversion of sputum at 2 months of anti-TB treatment and the time to culture conversion were different among pulmonary TB patients diagnosed by different methods. Culture-confirmed pulmonary TB patients who had negative results on AFB smears or were diagnosed using bronchoscopic specimens showed higher culture conversion rates at 2 months of treatment and a shorter time to negative conversion than those with smear-positive pulmonary TB.

Previous studies have reported that the presence of cavities [14], involvement of bilateral lung [15], and high pre-treatment smear grade [13] are associated with delayed culture conversion. We also showed that patients with smear-positive pulmonary TB had delayed culture conversion compared with those with smear-negative pulmonary TB or TB patients diagnosed using bronchoscopic specimens. The association between delayed culture conversion and the presence of cavities, bilateral lung involvement, and smear positivity could be due to a high bacillary burden. Having a high bacillary burden implies stronger infectivity and requires a longer isolation period and more intensive treatment [16]. Based on our finding that pulmonary TB patients who had negative results on a smear or those who were diagnosed using bronchoscopic specimens showed a shorter time to negative conversion than those with smear-positive pulmonary TB, the duration of isolation could be shorter than the conventionally recommended 2 weeks for TB patients [17].

In treatment of pulmonary TB, culture conversion status at 2 months of treatment is known to be associated with relapse [8]. According to a recent analysis, new regimens of 4 or 5 months' duration with rates of culture positivity after 2 months of 1% or 3% would yield relapse rates of 4.0% or 4.1%, respectively [18]. In our study, the culture conversion rate at 2 months was 94.1% in the culture-positive group and 97.6% in the bronchoscopy group, but only 78.7% in the smear-negative group. These results suggest that a regimen shorter than 6 months could be adopted for TB patients with AFB smear-negative sputum. In fact, several previous studies have reported that a 4-month regimen is a reliable treatment option for smear-negative pulmonary TB [19]-[21], although it has not been adopted by official guidelines yet [2], [22], [23].

This study has several limitations. First, sputum collection times were not uniform in every patient included in this study. Although sputum culture results between 8 and 9 weeks of treatment were available in all of the patients included in this analysis, monthly cultures were lacking in some patients. This could have caused overestimation of the time to culture conversion. Second, TB patients who could not expectorate sputum after the initiation of treatment were excluded. This might also have caused overestimation of the time to culture conversion, as well as underestimation of culture conversion rates. Third, the time to culture conversion might have been slightly different from the ideal time because it could have been affected by the intervals between mycobacterial cultures of sputum. Lastly, we defined culture conversion based on liquid culture. This prevents a direct comparison with previous studies on culture conversion using solid media because detection using an automated continuous monitoring system with liquid medium can detect a higher amount of *M.tuberculosis* and is more rapid than using a solid medium[24].

## Conclusion

Smear-negative pulmonary TB patients have a higher culture conversion rate at 2 months of treatment and a shorter time to negative conversion than those with positive AFB staining of sputum. This observation suggests the feasibility of a shorter treatment and shorter isolation period for smear-negative pulmonary TB patients than for smear-positive pulmonary TB patients.
